# Cytoplasmic Localization of p21 Protects Trophoblast Giant Cells from DNA Damage Induced Apoptosis

**DOI:** 10.1371/journal.pone.0097434

**Published:** 2014-05-21

**Authors:** Christelle de Renty, Melvin L. DePamphilis, Zakir Ullah

**Affiliations:** 1 Eunice Kennedy Shriver National Institute of Child Health and Human Development, National Institutes of Health, Bethesda, Maryland, United States of America; 2 Department of Biology, School of Science and Engineering, Lahore University of Management Sciences, Lahore, Pakistan; Sanford Burnham Medical Research Institute, United States of America

## Abstract

Proliferating trophoblast stem cells (TSCs) can differentiate into nonproliferating but viable trophoblast giant cells (TGCs) that are resistant to DNA damage induced apoptosis. Differentiation is associated with selective up-regulation of the Cip/Kip cyclin-dependent kinase inhibitors p57 and p21; expression of p27 remains constant. Previous studies showed that p57 localizes to the nucleus in TGCs where it is essential for endoreplication. Here we show that p27 also remains localized to the nucleus during TSC differentiation where it complements the role of p57. Unexpectedly, p21 localized to the cytoplasm where it was maintained throughout both the G- and S-phases of endocycles, and where it prevented DNA damage induced apoptosis. This unusual status for a Cip/Kip protein was dependent on site-specific phosphorylation of p21 by the Akt1 kinase that is also up-regulated in TGCs. Although cytoplasmic p21 is widespread among cancer cells, among normal cells it has been observed only in monocytes. The fact that it also occurs in TGCs reveals that p57 and p21 serve nonredundant functions, and suggests that the role of p21 in suppressing apoptosis is restricted to terminally differentiated cells.

## Introduction

Genes that regulate cell proliferation and differentiation during mammalian development are often deregulated in human cancer, thereby permitting either unrestrained cell proliferation or increased survivability. One example is the phosphatidylinositol-3 kinase (PI3K) signal transduction pathway that influences cell proliferation, survival, metabolism, and metastasis [1]. Alterations in upstream components of the PI3K pathway, such as receptor tyrosine kinases, and downstream components such as the Akt/PKB serine/threonine kinase family are frequently found in cancers [2]. Akt/PKB is activated by growth factors and promotes cell survival by preventing apoptosis [3]. Of particular interest is the fact that Akt1 phosphorylates the human Cdkn1A/p21/Cip1 (p21) protein at T145 and S146, thereby stabilizing the protein and localizing it to the cytoplasm [4]–[8]. The p21 protein is one of the three Cip/Kip cyclin-dependent kinase (CDK) inhibitors that commonly localize to the nucleus where they regulate cell proliferation and differentiation [9]. However, Akt1-dependent cytoplasmic localization of p21 occurs in a variety of cancers where it promotes tumorigenesis by inhibiting proteins essential for apoptosis [5]–[7], [10]–[14].

Remarkably, during normal mammalian development, cytoplasmic localization of p21 has been reported only in monocytes where it promotes resistance to apoptosis [15], [16]. Such rarity confirms that the PI3K/AKT/mTOR pathway must be highly regulated in order to protect cells from premature senescence or from producing a cancer [17]. Given that monocytes simply migrate into tissues where they differentiate further into macrophages, we considered the possibility that cytoplasmic localization of p21 is restricted to terminally differentiated cells that no longer proliferate. Such a mechanism would be particularly beneficial to cells undergoing multiple S-phases in the absence of an intervening mitosis and cytokinesis (termed endoreplication or endomitosis [18]), because such cells are more likely to accumulate stalled replication forks and DNA damage.

Here we show that Akt-dependent cytoplasmic localization of p21 and its ability to suppress DNA damage induced apoptosis also occurs when trophoblast stem cells (TSCs) differentiate into trophoblast giant cells (TGCs). The rapidly proliferating TSCs exit their mitotic cell cycle in response to environmental signals and initiate multiple rounds of endoreplication (termed ‘endocycles’) to produce the nonproliferating, polyploid, viable TGCs that are essential for embryo implantation and placental development [19]. During normal preimplantation mouse development, maintenance of the undifferentiated trophoblasts that constitute the outer epithelial layer of a blastocyst depends on the presence of fibroblast growth factor-4 (FGF4) and perhaps other mitogens that are essential for trophoblast cell proliferation [20]–[24]. Differentiation of trophoblast cells into TGCs first occurs during implantation when trophoblasts in the mural trophectoderm are deprived of FGF4 [25], a phenomenon that is recapitulated *in vitro* by TSCs.

TSCs are derived from the trophectoderm of the blastocyst and give rise exclusively to all of the trophoblast lineages in the placenta [26]–[28]. TSCs proliferate in the presence of FGF4 and medium conditioned by preincubation with primary mouse embryonic fibroblasts, but in their absence, TSCs initiate a sequence of events that culminates in the expression of p21 and Cdkn1C/p57/Kip2 (p57) [29]–[31], another member of the Cip/Kip family of CDK inhibitors. Expression of p57 and p21 is regulated post-translationally by the Chk1 kinase; Chk1 phosphorylation of p57 and p21 in TSCs targets them for ubiquitin dependent degradation, whereas down-regulation of Chk1 protein in TGCs allows expression of p57 and p21 [32]. Expression of Cdkn1B/p27/Kip1 (p27), the third Cip/Kip member, remains comparatively constant during TSC differentiation. The nuclear localization and role of p57 in the initiation and maintenance of endoreplication cycles has been well documented [30], [31], [33]. The role of p27 has not been explored, because its expression remains unchanged during TSC differentiation. A role for p21 in preventing apoptosis has been suggested [31], but it remains to be established.

The p27 and p57 proteins are essential for normal mouse development only after midgestation [34], but embryos deficient in p21 develop normally and without spontaneous tumors to produce viable fertile adults [35]. Thus, the multiple regulatory roles for which p21 has been implicated in cell proliferation, migration, apoptosis, senescence, and differentiation [36] are auxiliary roles that facilitate the activities of other genes. Furthermore, the role played by p21 depends on whether it is in the nucleus or the cytoplasm. Nuclear p21 inhibits cell proliferation in response to DNA damage or replication stress through inhibition of Cdk1 and Cdk2 [37], [38] and by binding to proliferating cell nuclear antigen (PCNA), an auxiliary component of DNA polymerases δ and ε [39]. Cytoplasmic p21 suppresses apoptosis in cancer cells and monocytes. Here we extend this discovery to include TGCs, and we demonstrate that cytoplasmic localization of p21 in TGCs is driven by site-specific phosphorylation of p21 protein by the Akt1 kinase.

## Results

### The p27 And p57 Proteins are Localized to the Nuclei Of G-Phase TGCs

**Figure 1:**
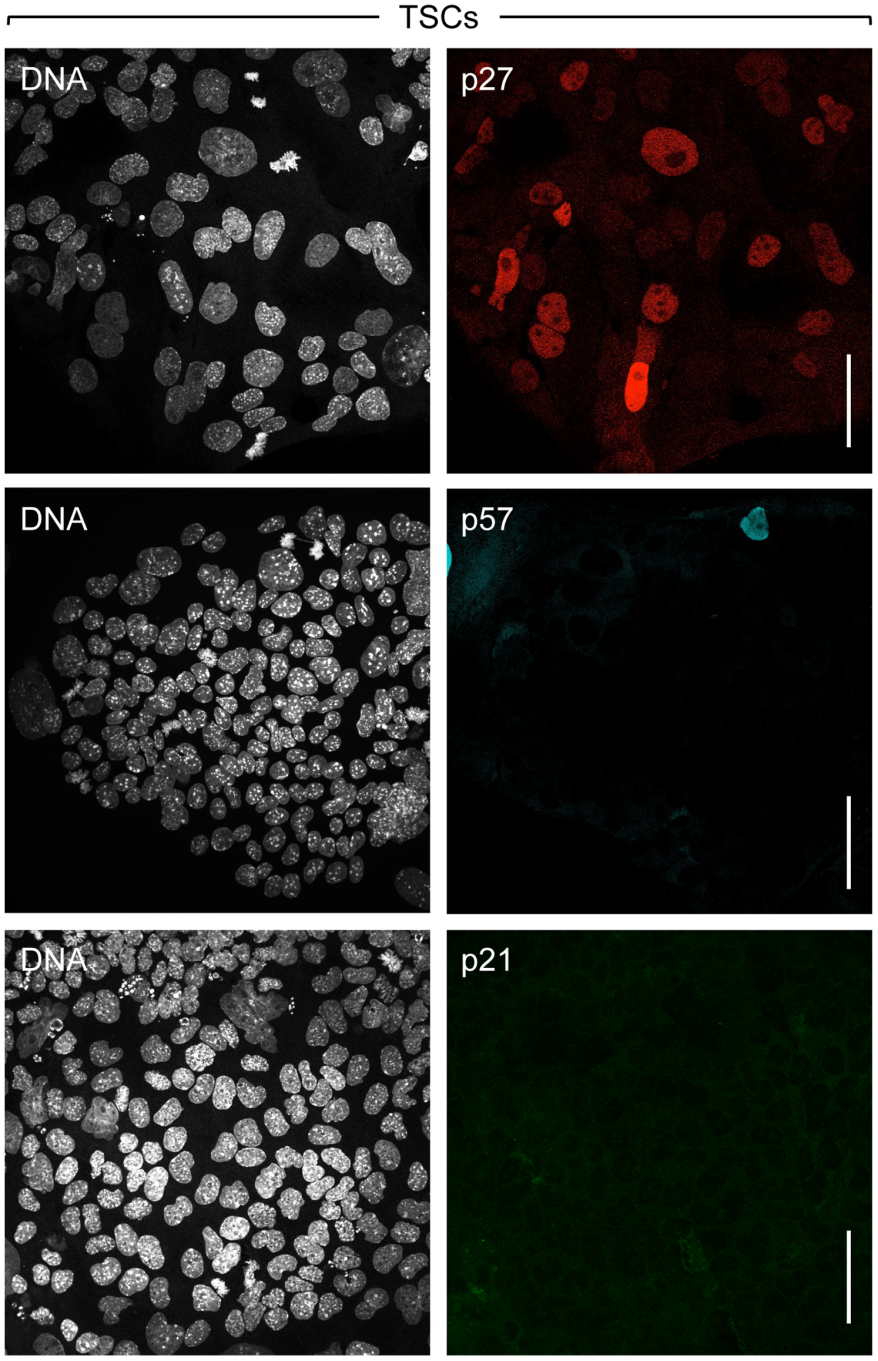
Only the Cip/Kip p27 protein is expressed in TSCs. Proliferating TSCs were fixed, stained for nuclear DNA (Hoechst 33342), and stained with antibodies against p21, p27 or p57 proteins. Images were acquired with a confocal microscope (63x objective). Scale bars represent 50 µm.

**Figure 2:**
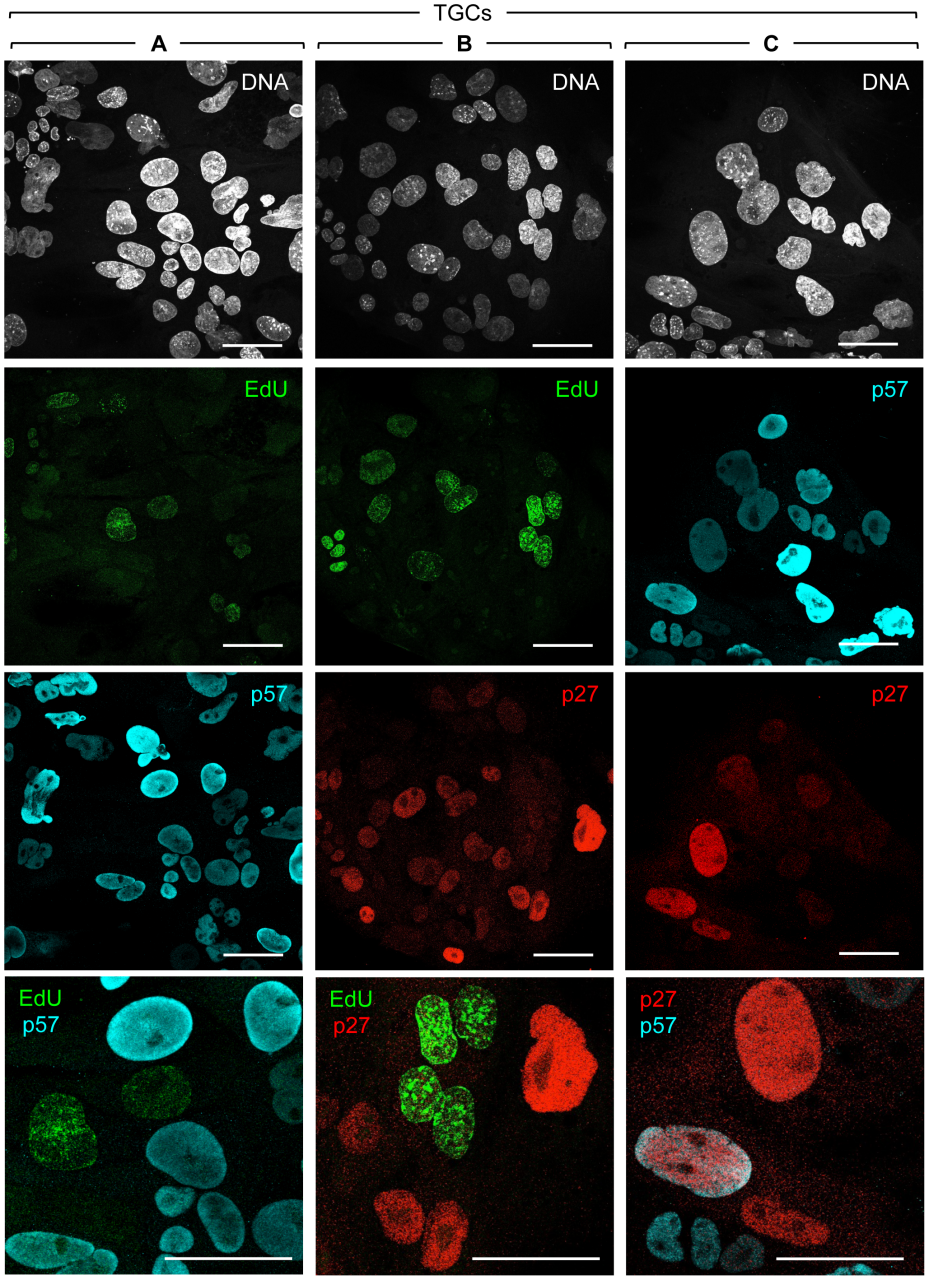
The p27 and p57 proteins are localized to the nucleus in G-phase TGCs. After three days of FGF4 deprivation, wild-type TGCs were cultured for 20 min in the presence of EdU (10 µM) to label S-phase cells. After fixation, EdU was detected using Click-iT chemistry (green), and the cells were stained with either anti-p57 (cyan) (A, C) or anti-p27 (red) (B, C) antibodies, and with Hoechst 33342 to visualize nuclear DNA (gray). Images were acquired with a confocal microscope (63x objective). The bottom panels are higher magnifications of merged images in order to visualize differential staining of EdU, p27 and p57. Scale bars represent 50 µm.

Based on RT-PCR and Western immuno-blotting analyses, previous studies have shown that all three Cip/Kip genes are transcribed in both TSCs and TGCs, but the p21 and p57 proteins are expressed primarily, if not exclusively, in TGCs [30], [31], [33]. In marked contrast, p27 protein is expressed at comparable levels in both TSCs and TGCs. Previous studies also have shown that p27 protein is localized to the nucleus in proliferating mammalian cells [40] where it prevents premature initiation of S-phase by inhibiting CcnE•Cdk2 activity during G1-phase [41]. Consistent with those studies, p27 protein was localized to the nucleus in both TSCs (Fig. 1) and TGCs (Fig. 2B, C).

In contrast with p27, only trace amounts of p57 protein were detected in TSC populations (Fig. 1), and this resulted from a small fraction of TSCs that differentiated spontaneously into TGCs [29]. As previously reported, the p57 protein was detected in TGCs only during G-phase where it was localized to the nucleus (Fig. 2A) [31], [33]. The fraction of TGC nuclei expressing p57 was in proportion to the fraction of G-phase cells (Table 1), consistent with a role for p57 in suppressing CDK activity and thereby preventing TGCs from initiating either DNA replication or mitosis. To determine whether or not p27 expression in TGCs is also restricted to G-phase nuclei, newly synthesized DNA in TGCs was labeled briefly by incorporation of EdU. Both p27 and p57 proteins were detected only in G-phase cells (Fig. 2A; B). Furthermore, p27 and p57 proteins were generally localized in the same nucleus, although in some cells either p27 or p57 alone was evident in G-phase nuclei.

**Table 1:** Expression of p21, p27 and p57 in TSCs and TGCs.

Cells	p21 protein	p27 protein	p57 protein	G phase	S phase	M phase
TSCs	<1%	31%	3.5%	36%	60%	3.5%
TGCs	100%	47%	40%	74%	26%	<1%

**Figure 3:**
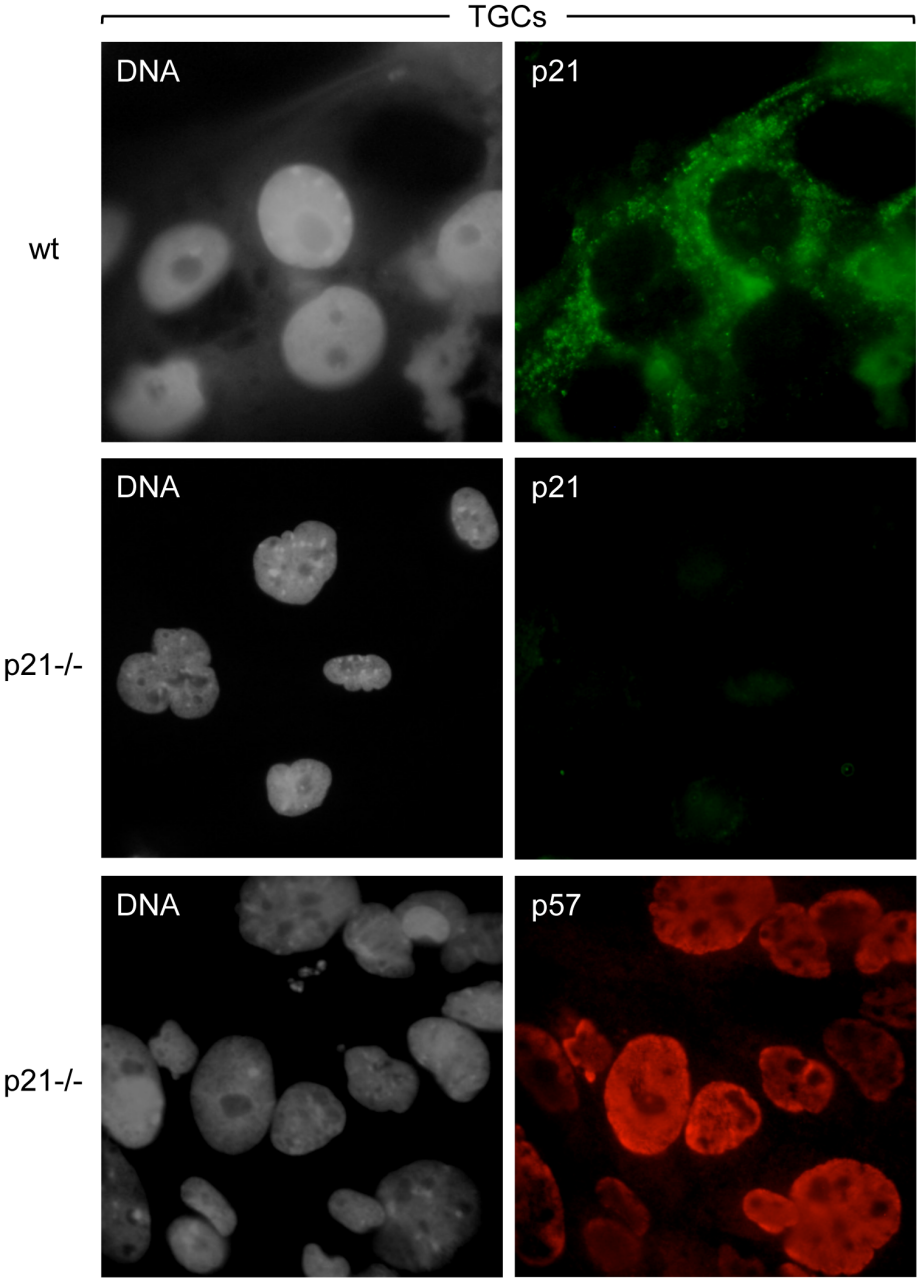
The p21 protein localized to the cytoplasm in TGCs. After three days of FGF4 deprivation, wild-type and *p21−/−* TGCs were stained for nuclear DNA (gray), p21 (green) and p57 (red), as in figure 1. The *p21−/−* TGCs expressed nuclear p57 protein, but not cytoplasmic p21 protein. Images were acquired with an epifluorescence microscope (60x objective). The same exposure time was used for wild-type and *p21*−/− TGCs acquisitions.

**Figure 4:**
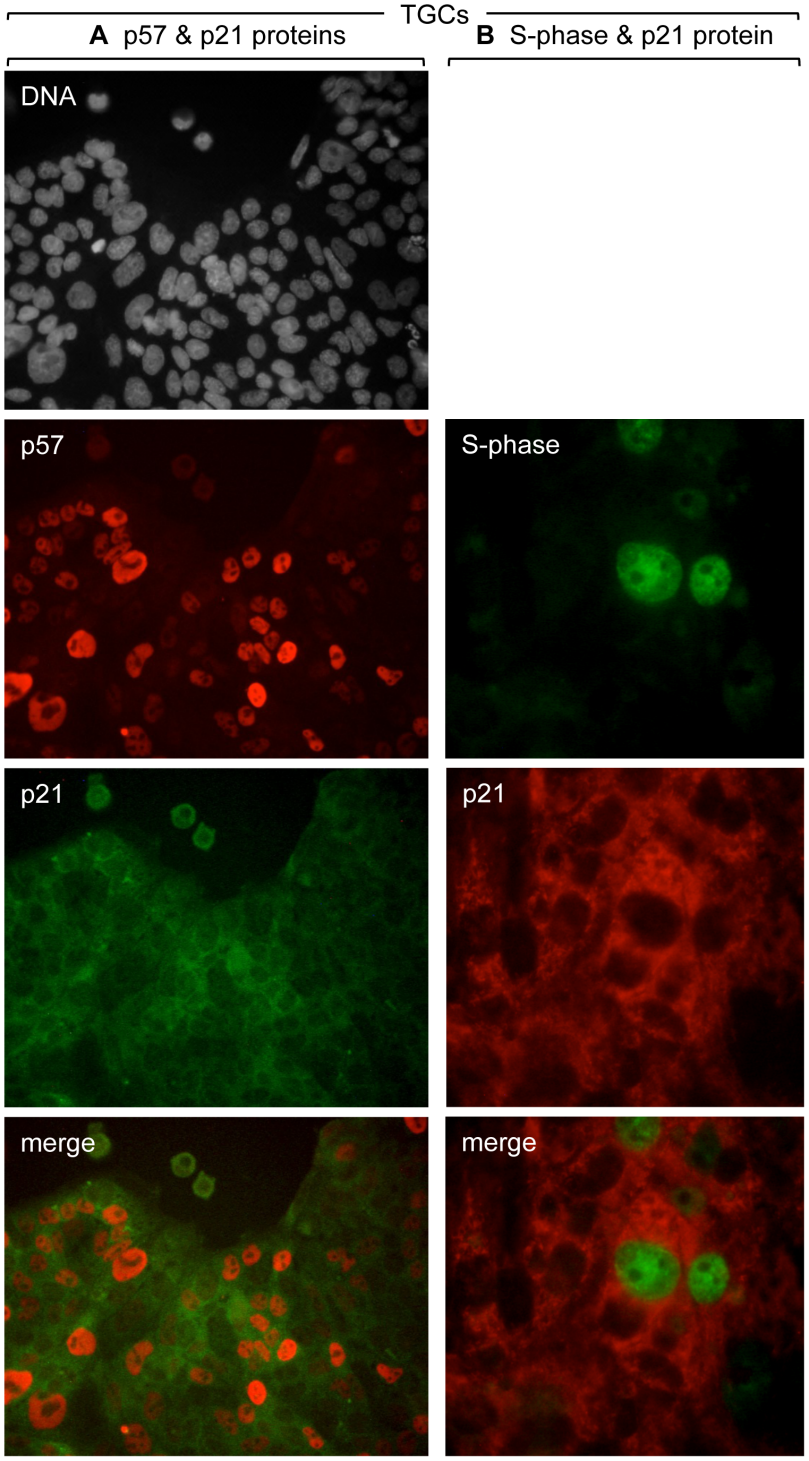
The p21 protein localized to the cytoplasm of both S-phase and G-phase TGCs. (A) Wild-type TGCs were prepared as in figure 2 and stained for nuclear DNA (gray), p57 (red), and p21 (green). Images were acquired with an epifluorescence microscope using a 40x objective. (B) The same TGCs were also cultured for 30 min with BrdU (10 µM). Following cell fixation and DNA denaturation, the cells were stained with anti-BrdU (green) and anti-p21 (red) antibodies. Images were acquired with an epifluorescence microscope with a 60x objective.

Cells were stained for the indicated protein, or for DNA, or for incorporation of EdU (S-phase cells) as described in Figures 1–4. M-phase cells were identified by the presence either of condensed chromatids or of mitotic figures. TGCs were examined three (3) days after FGF4 deprivation of TSCs. A minimum of 100 cells was scored in each case in order to determine the fraction of cells undergoing the indicated event. %G phase cells = 100– (%S phase cells+%M phase cells).

In some TGCs, EdU, anti-p27 and anti-p57 either did not stain the nucleus or stained the nucleus only lightly (Fig. 2). This reflects the fact that endoreplication occurs asynchronously within this population of cells. Cells either entering or leaving G-phase would be expected to contain reduced levels to less than detectable levels of DNA synthesis and G-specific proteins. Nevertheless, detectable levels of either EdU and p57, or EdU and p27 in the same nucleus were not observed, whereas detectable levels of p27 and p57 in the same nucleus were observed (Fig. 2, bottom panels). When quantified (Table 1), the proportion of nuclei containing detectable levels of either p27 or p57 protein was proportional to the fraction of G-phase cells. These results were consistent with the established role of p27 in preventing premature onset of S-phase, as well as the fact that p27 cannot substitute for p57 in placenta development [42].

### The p21 Protein is Localized to the Cytoplasm of Both G and S-Phase TGCs

As expected from Western immuno-blotting analyses [30], [31], p21 protein was not detected in TSCs by immuno-fluorescence (Fig. 1), but it was detected in TGCs. However, in contrast to the nuclear localization of p27 and p57 proteins in TGCs, p21 protein localized exclusively to the cytoplasm (Fig. 3). This result was confirmed by the fact that p21 protein was not detected by immuno-staining of *p21−/−* TGCs, whereas p57 protein was readily detected in the nuclei of *p21−/−* TGCs. A second distinction between p21 and p57 was that p21 protein was present throughout the TGC population, whereas p57 protein was present only in the nuclei of G-phase cells. Immuno-staining of wild-type TGCs, in which replicating DNA had been pulse-labeled with BrdU, confirmed that p21 protein was present in the cytoplasm of S-phase as well as G-phase cells (Fig. 4).

### Phosphorylation at the p21 Akt1/NLS Stabilizes p21 Protein in TGCs

**Figure 5:**
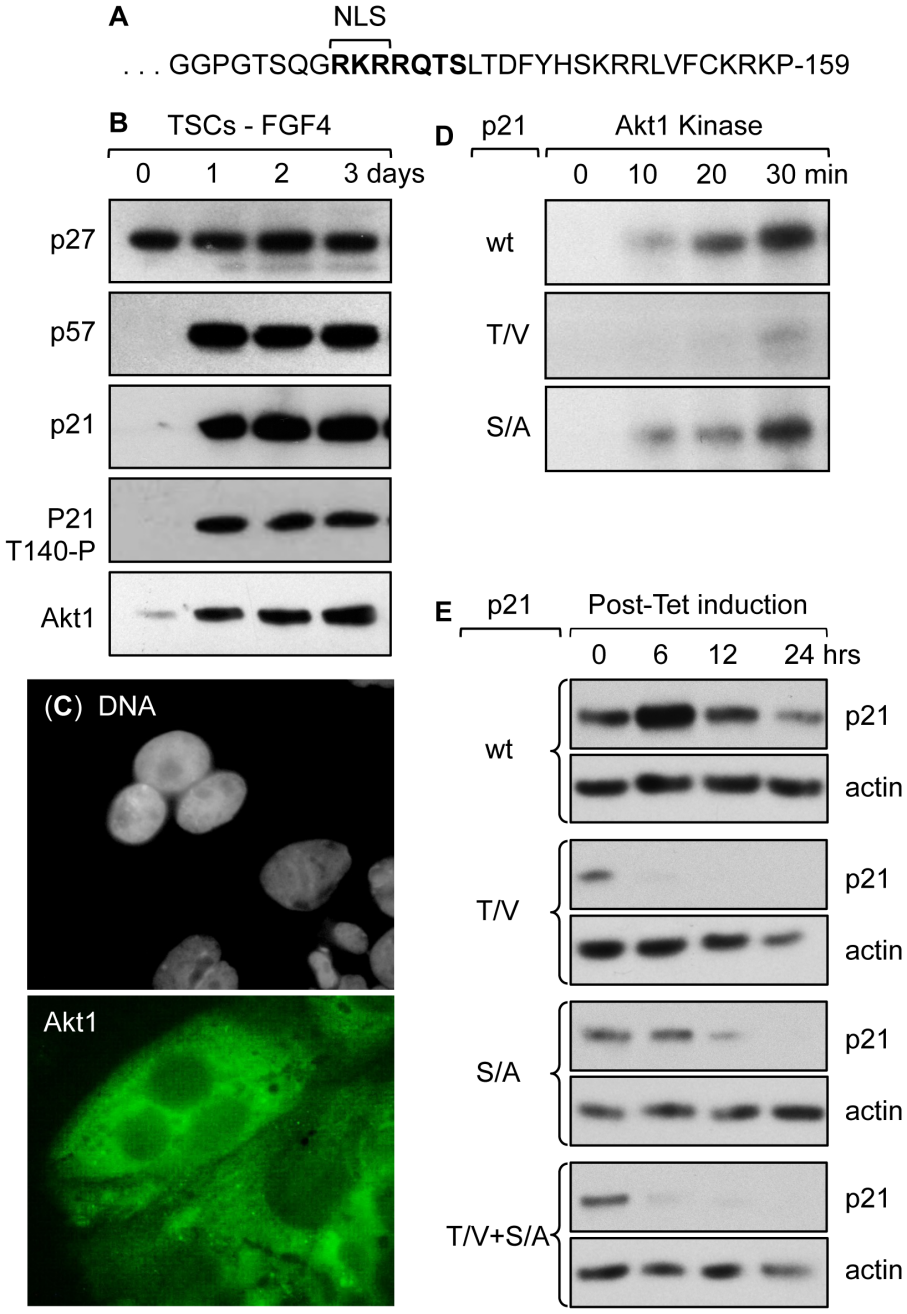
Akt1 is required for p21 stabilization in TGCs, and Akt1 phosphorylates p21 at T140. (A) C-terminal sequence of mouse p21 protein with the putative Akt1 phosphorylation site (RKRRQTS) and the essential RKR nuclear localization signal [44] in bold face. (B) Extracts of TSCs and TGCs were assayed by Western immuno-blotting using antibodies specific for Akt1, p21, p27 and p57 proteins. The phosphorylated form of p21 was recognized as a p21 protein that migrated slower than unphosphorylated p21, and by its reaction with an anti-p21 antibody specific for Thr-145 phosphorylation in human p21. (C) After three days of FGF4 deprivation, TGCs were stained with 4,6-diamidino-2-phenylindole (DAPI) to visualize nuclear DNA (gray) and with anti-Akt1 antibody (green). (D) Wild-type p21 and two p21 mutant forms with a T140V or a S141A substitution were tested as substrates for phosphorylation by Akt1 *in vitro*. (E) NIH3T3 fibroblasts were co-transfected with a plasmid expressing the tetracycline repressor and a plasmid encoding the indicated p21 protein whose expression was regulated by a tetracycline inducible promoter. Each protein carried a [His]_6_-cMyc-epitope tag fused to its C-terminus. After 24 hours of transfection, the cells were cultured for 18 hours in the presence of tetracycline (1 µg/ml) in order to induce expression of the indicated recombinant p21 protein. Cells were then harvested at 0, 6, 12 and 24 hours after release, and extracts were analyzed for the indicated protein by Western immuno-blotting using a Myc-Tag specific antibody. Wild-type (wt) p21 and the T140V (T/V) and S141A (S/A) p21 mutants, as well as a double mutant (T/V+S/A) were tested. Actin served as a loading control in each case.

The mouse p21 protein, like its human homolog, contains a single putative Akt1 consensus phosphorylation site (RxRxxT/S [43]) coincident with its nuclear localization signal [44] near the C-terminus (RKRRQTS-141, Fig. 5A). Therefore, we reasoned that the Akt1 kinase might stabilize mouse p21 protein in TGCs and localize it to the cytoplasm, as previously demonstrated in human cancer cells [5]–[7], [10]–[14]. In fact, Akt1 expression was up-regulated in TGCs concomitant with up-regulation of p21 and p57 expression (Fig. 5B), and Akt1 protein, like p21 protein, was localized to the cytoplasm (Fig. 5C). Moreover, Western immuno-blotting with phospho-specific antibodies raised against the Akt1 phosphorylation consensus site in p21 indicated that Akt1 phosphorylated p21 in TGCs (Fig. 5B).

To determine whether or not Akt1 can phosphorylate mouse p21 at T140, recombinant p21 proteins were tested as Akt1 substrates *in vitro* (Fig. 5D). The results showed that wild-type (wt) p21 protein was phosphorylated primarily at T140, since site-specific substitution of valine for threonine 140 (T140V) greatly reduced the ability of Akt1 to phosphorylate p21. In contrast, site-specific substitution of alanine for serine 141 (S141A) only marginally reduced the ability of mouse p21 to serve as a substrate for Akt1. Nevertheless, subsequent analysis of p21 stability *in vivo* revealed the importance of both T140 and S141 phosphorylation.

The stability of mouse p21 protein *in vivo* was evaluated by transfecting mouse NIH3T3 cells, an immortalized cell line derived from mouse primary embryonic fibroblasts, with plasmids that expressed cMyc-tagged p21 proteins under control of a tetracycline inducible promoter. Previous studies demonstrated that NIH3T3 cells mimic the response of trophoblasts to elevated levels of p57 protein, and they have the experimental advantage being transfected more efficiently than trophoblast cells [30]. Transfected cells were allowed to accumulate the recombinant p21 protein by culturing them in the presence of tetracycline for 18 hours. Cells were then cultured in tetracycline-free medium, and p21 stability was analyzed during the following 24 hours. The results revealed that p21(wt) protein was stable under conditions where p21(T140V), p21(S141A), and p21(T140V+S141A) proteins were degraded (Fig. 5E). p21(S141A) was clearly less stable than p21(wt), but it was significantly more stable than p21(T140V). p21(S141A) was detected up to 12 hours after removal of tetracycline, whereas p21(T140V) could not be detected by 6 hours after removal of tetracycline. Taken together, these results demonstrated that the Akt1 kinase phosphorylates mouse p21 protein *in vivo*, and that this phosphorylation event inhibits p21 degradation.

### Phosphorylation Of p21 at the Akt1/NLS Targets p21 to the Cytoplasm

**Figure 6:**
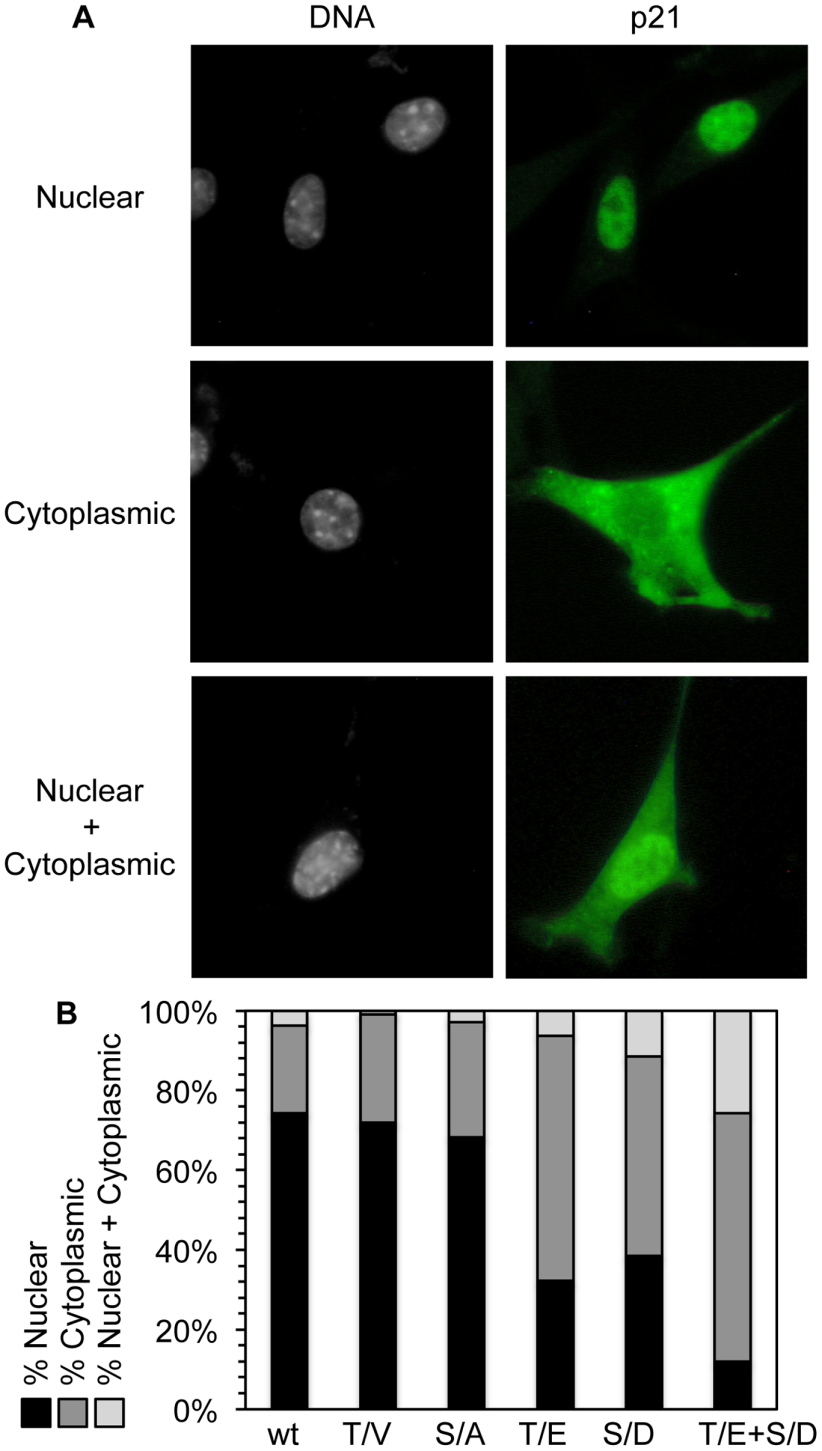
Phosphomimetic mutations in the Akt1 phosphorylation site induced cytoplasmic localization of p21. (A) Representative immuno-fluorescence images (60x objective) of ectopically expressed p21 protein 24 hours post-transfection of NIH3T3 cells using anti-Myc-Tag antibody illustrate nuclear, cytoplasmic, and nuclear plus cytoplasmic p21 localization. (B) Recombinant mouse p21 proteins were expressed in NIH3T3. Localization of the indicated p21 protein was detected by immuno-staining with an anti-Myc-Tag antibody, 24 hours after transfection. In addition to wild-type (wt) p21 and the T140V (T/V) and S141A (S/A) p21 mutants, T140E (T/E) and S141D (S/D) p21 phosphomimetic mutants, and a double phosphomimetic mutant (T/E+S/D) were also tested. More than 100 cells were scored for each transfection.

To determine whether or not Akt1-mediated phosphorylation localizes p21 to the cytoplasm in TGCs, the subcellular localization of the p21 mutants described in figure 5E was characterized. Based on immuno-staining with anti-Myc antibody, the cMyc-p21 protein in transfected NIH3T3 cells was classified as nuclear, cytoplasmic, or both nuclear and cytoplasmic (Fig. 6A). At least 70% of the cells that ectopically expressed p21(wt), p21(T140V) or p21(S141A) localized these proteins to the nucleus (Fig. 6B). However, when either T140 was changed to glutamic acid (T140E), or S141 was changed to aspartic acid (S141D) in order to mimic the phosphorylated state of threonine and serine, respectively, then greater than 50% of the ectopically expressed p21 protein was localized to the cytoplasm. When both T140 and S141 were converted into their phosphomimetic forms, then the modified protein was nuclear in only 12% of the cells, cytoplasmic in 63% of the cells, and both nuclear and cytoplasmic in 25% of the cells. Thus, the number of cells in which ectopically expressed p21 was localized to the cytoplasm was 3-times greater for p21(T140E+S141D) than for p21(wt). These data, together with those in figure 5, revealed that Akt1 phosphorylation of both T140 and S141 in p21 stabilizes the protein and localizes it to the cytoplasm.

### p21 Facilitates TGC Resistance to DNA Damage

**Figure 7:**
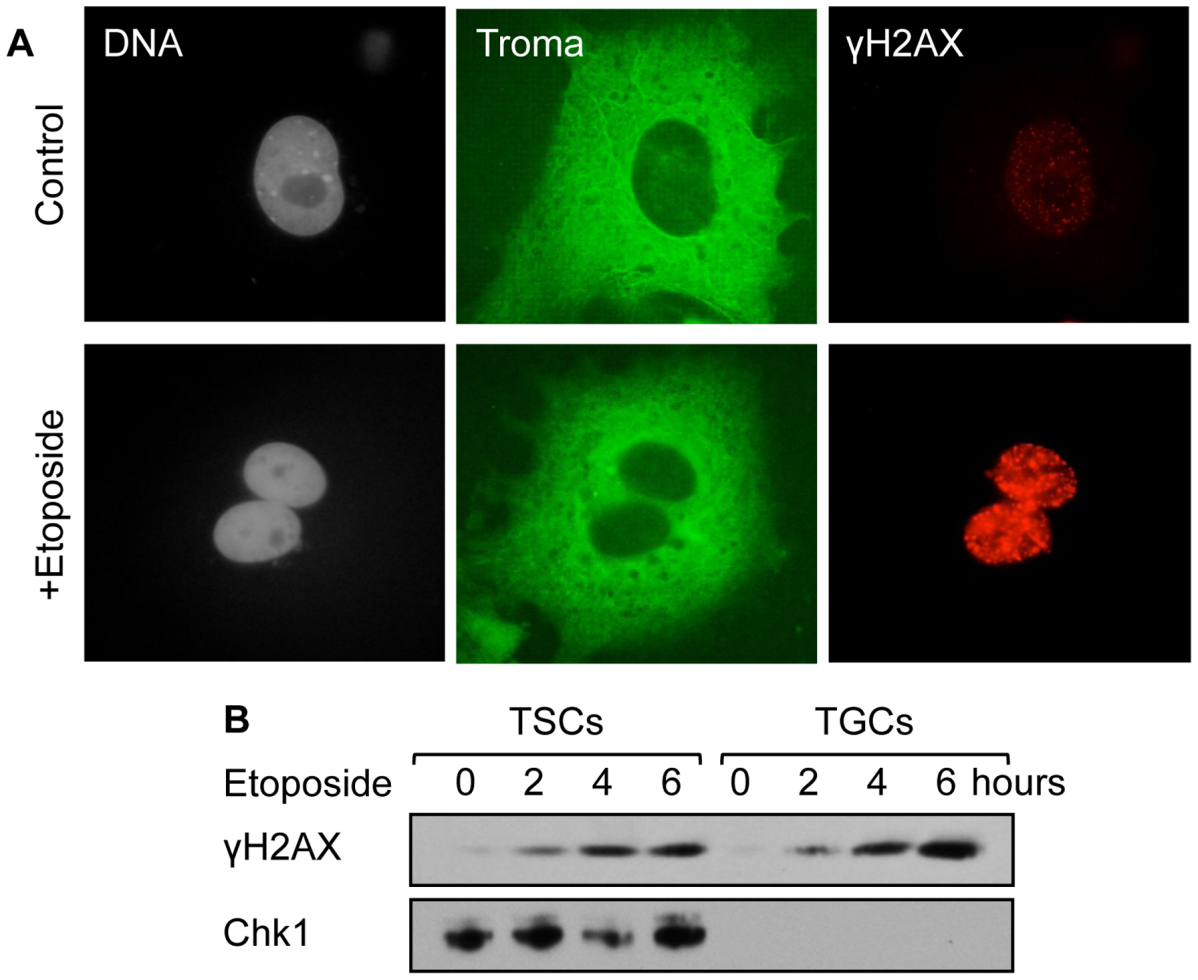
DNA damage can be induced in TGCs in the absence of the Chk1 kinase. (A) Wild-type TGCs at three days post-FGF4 deprivation (control) were treated with 5 µM etoposide (+ Etoposide) for 3 days. Cells were stained with DAPI to visualize nuclear DNA (gray), Troma-1 antibody to visualize the cytoplasm (green), and anti-γH2AX to visualize areas where double stranded DNA breaks occurred (red). (B) Wild-type TSCs and TGCs three days after FGF4 deprivation were treated with 5 µM etoposide for the indicated times. Chk1 protein and histone H2AX (γH2AX) phosphorylation were assayed by western immuno-blotting.

Etoposide stabilizes a covalent complex between topoisomerase II and DNA. Collisions of DNA replication forks with these complexes convert them into double-strand DNA breaks that trigger apoptosis [45]. Double-strand DNA breaks induce phosphorylation of histone H2AX that can be detected with antibodies specific for the phosphorylated product γH2AX [46]. As expected, addition of etoposide to TGC culture medium induced the appearance of γH2AX in TGC nuclei (Fig. 7A) as well as in Western immuno-blots of TGC extracts (Fig. 7B). Thus, DNA damage accumulates in TGCs in the absence of the Chk1 kinase (Fig. 7B; [31], [32]).

**Figure 8:**
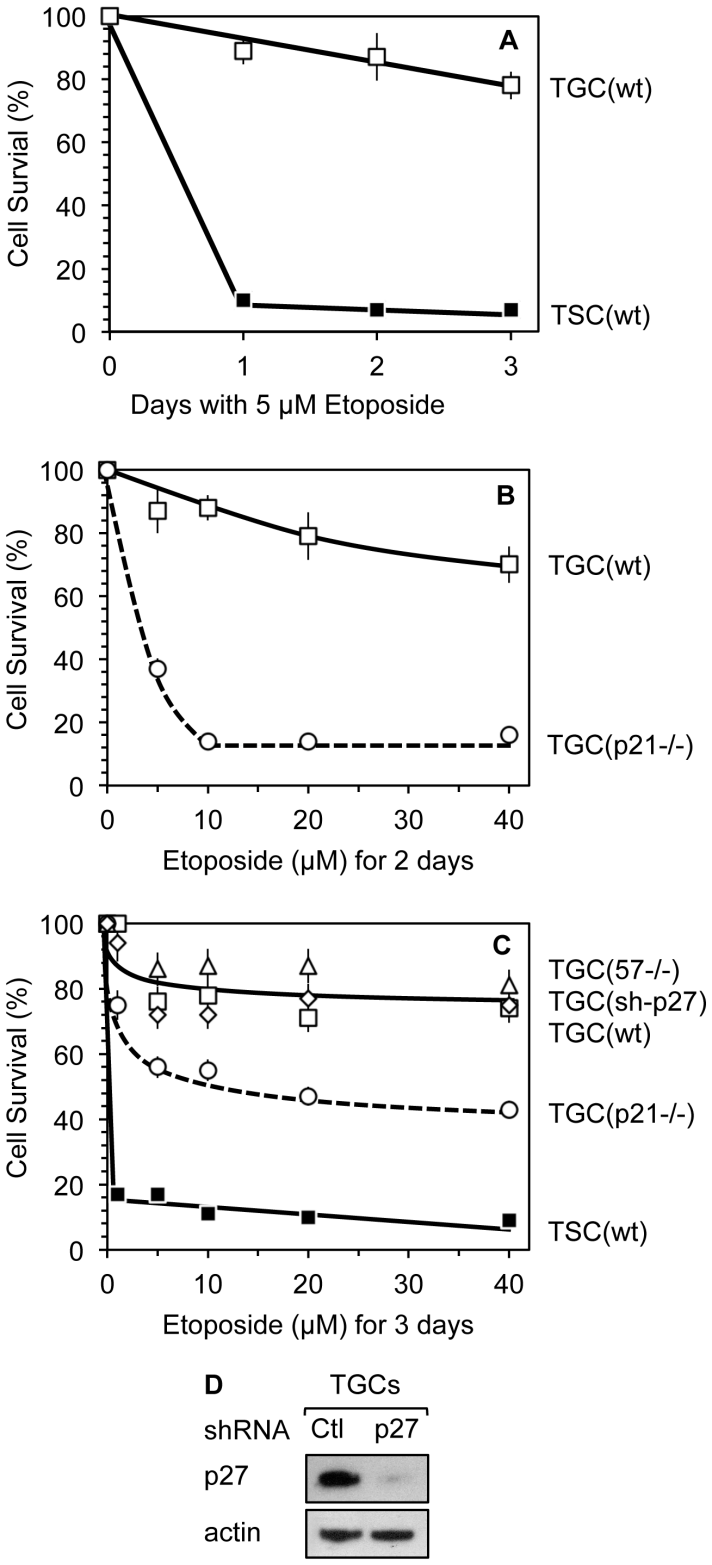
Cytoplasmic p21 provided TGCs with resistance to DNA damage induced apoptosis. (A) Wild-type TSCs (▪, 6×10^5^ cells), cultured in standard TSC medium, were treated with 5 µM etoposide for up to three days. Wild-type TGCs (□, 6×10^5^ cells) were produced by FGF4 deprivation of TSCs for three days, 5 µM etoposide was then added to the medium for up to three days. The number of cells remaining attached to the dish was recorded and the results calculated as the percentage of cells that survived. (B) Same as in A except that wild-type TGCs (□) and *p21−/−* TGCs (○, broken line) had been deprived of FGF4 for only two days before treating them with the indicated concentration of etoposide for two days. Wild-type and *p21−/−* TSCs were indistinguishable during their proliferation and their differentiation into TGCs. (C) Wild-type TSCs (▪) and TGCs (□), cultured as in panels A and B, were exposed to indicated concentration of etoposide for three days and cell survival was assessed as in B. Wild-type TGCs infected with lentivirus containing shRNA against p27 (⋄), *p57−/−* TGCs (▵) and *p21−/−* TGCs (○, broken line) were treated as well. (D) Depletion of p27 protein in TGCs treated either with shRNA against p27 RNA or scramble control shRNA (Ctl) was assayed by Western immuno-blotting. Errors bars indicate standard error of the mean for triplicate samples of each datum point.

However, compared to TSCs, TGCs were resistant to apoptosis induced by DNA damage. For example, within one-day 5 µM etoposide killed 90% of TSCs, but only 10% of TGCs (Fig. 8A). Notably, the resistance of TGCs to etoposide-induced apoptosis was dependent in large part on the presence of a functional *p21* gene (Fig. 8B). Two days after treatment with 10 µM etoposide, 70% of wild-type TGCs survived, whereas only 15% of *p21−/−* TGCs survived. In contrast to the effectiveness of p21 protein at reducing TGC sensitivity to etoposide-induced apoptosis, neither the suppression of p27 expression with shRNA nor the absence of *p57* gene (*p57−/−* TGCs) increased the sensitivity of TGCs to etoposide (Fig. 8C–D). Thus, etoposide rapidly killed TSCs with comparatively little effect on the viability of TGCs, and the resistance of TGCs to etoposide-induced apoptosis was reduced in the absence of *p21*. Similar results were obtained with staurosporine, another potent inducer of apoptosis [47], and with UV irradiation (data not shown).

### Akt1 Is Essential for Stabilizing Cip/Kip Proteins and for Preventing Apoptosis

**Figure 9:**
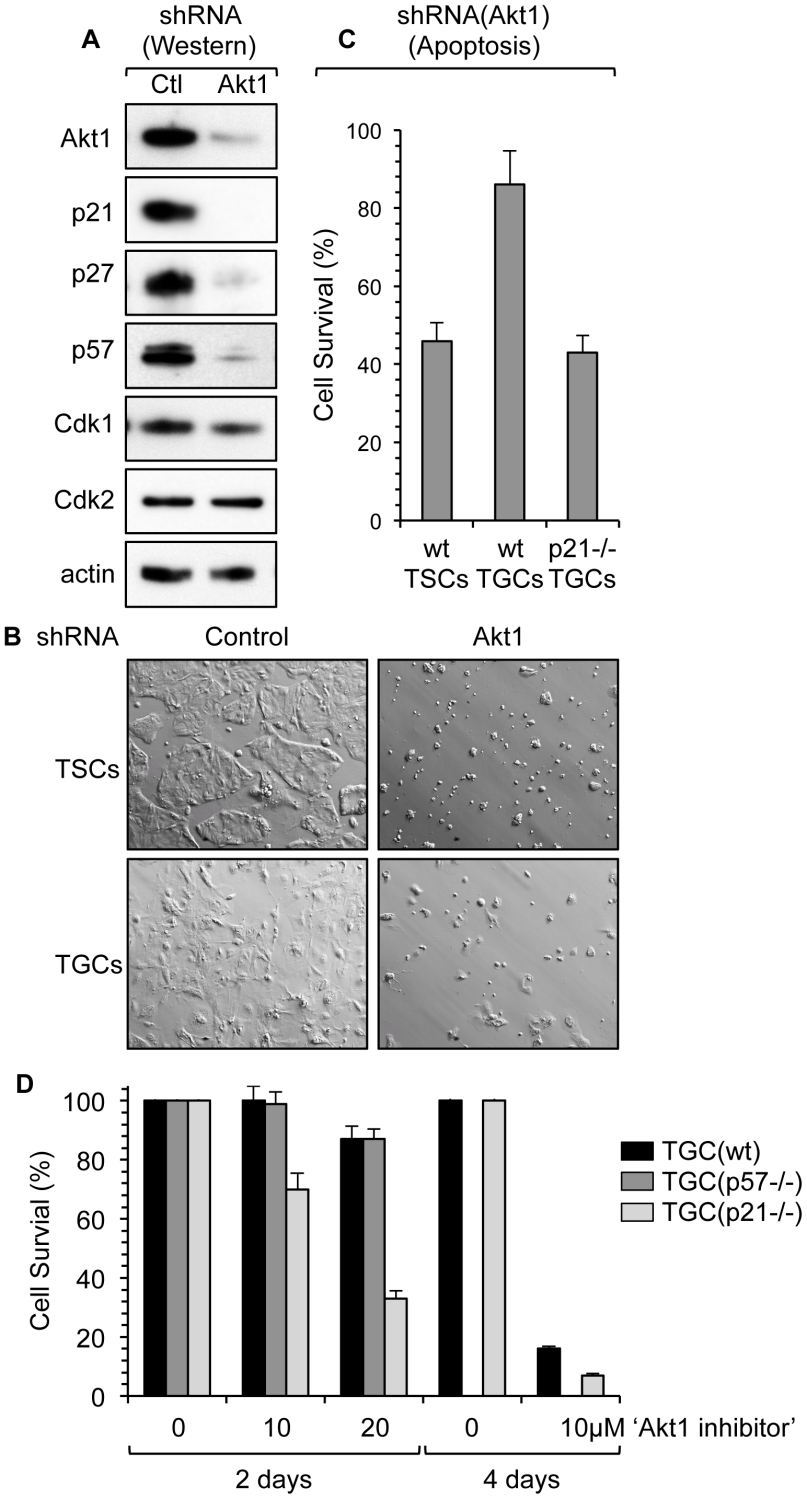
Inhibition of Akt1 activity induced apoptosis. (A) After three days of FGF4 deprivation, TGCs were transduced with lentiviruses expressing either control ‘scramble shRNA’ (Ctl) or shRNA targeted against Akt1. Two days after transduction, total cell lysates of surviving cells were analyzed by Western immuno-blotting for the indicated proteins. (B) DIC pictures of the cells three days after transduction (10x objective). (C) The fraction of cells remaining attached to the dish 2 days after lentivirus transduction are indicated for wild-type TSCs and TGCs, and for *p21−/−* TGCs. (D) TGCs 3 days post-FGF4 deprivation were treated with the indicated concentration of ‘Akt1 inhibitor-X’ (Santa Cruz) for the indicated period of time and cell survival calculated as in (C). Results are shown for wild-type TGCs (black bars), *p57−/−* TGCs (dark gray bars) and *p21−/−* TGCs (light gray bars). Error bars represent standard deviation.

Both the p21 and p57 proteins contain a single Akt1 consensus phosphorylation site, and murine p27 is phosphorylated by Akt1 at a nonconventional site [48]. Therefore, to determine whether or not Akt1 was essential for the stability of all three Cip/Kip proteins in TGCs, lentivirus was used to introduce DNA expressing shRNAs targeted against Akt1 RNA into TGCs. This not only resulted in a decrease in Akt1 protein levels, but the loss of p21, p27 and p57 proteins as well (Fig. 9A). However, neither the nuclear targets for these CDK specific inhibitors (Cdk1 and Cdk2), nor the cytoplasmic structural protein actin were suppressed, confirming the specificity of the shRNAs. These results revealed that Akt1 was required for the stability of all three Cip/Kip proteins in TGCs, although only Akt1-phosphorylated p21 protein localized to the cytoplasm. This distinction could result from the unique ability of the p21 nuclear localization signal to bind a cytoplasmic retention protein, such as Brap2, as reported in monocytes [16]. Brap2 is up-regulated concomitantly with p21 during monocytic differentiation [16], and genetic disruption of Brap2 in *C. elegans* impairs expression of p21 in response to oxidative stress [49].

Akt1 promotes cell proliferation and survival and counteracts apoptosis induced by anti-cancer drugs [50], [51]. Thus, it was not surprising that suppression of Akt1 expression either in TSCs, which do not express the p21 and p57 proteins, or in TGCs, which do express these proteins, resulted in cell death (Fig. 9B). However, TGCs were less sensitive to suppression of Akt1 than TSCs, a difference attributed to the presence of p21 protein in TGCs (Fig. 9C). The effects of shRNA against Akt1 on cell viability were confirmed by treating the cells with a specific inhibitor of Akt1 kinase (Fig. 9D). Treatment of wild-type TGCs for two days with 10 µM Akt1 inhibitor-X did not reduce cell viability, but treatment for 4 days reduced it 5-fold. However, *p21−/−* TGCs were twice as sensitive to inhibition of Akt1 activity as were wild-type TGCs, whereas *p57−/−* TGCs were as sensitive to inhibition of Akt1 activity as wild-type TGCs. Thus, Akt1 kinase activity is required for trophoblast viability, a role that is facilitated by p21.

## Discussion

The p21 protein is selectively up-regulated during differentiation of TSCs into TGCs [31] by suppression of the Chk1 kinase [30] and then stabilized in TGCs and localized to the cytoplasm through its site-specific phosphorylation by Akt1, a protein kinase that is also up-regulated during TSC differentiation (this report). Thus, p57 and p21 play distinctly different roles during the differentiation of TSCs into TGCs. Induction of p57 triggers endoreplication and sustains endocycles in the terminally differentiated TGCs, whereas induction of p21 suppresses apoptosis that would otherwise be induced by stalled replication forks or DNA damage.

### Genotoxic Stress

Compared to cells undergoing mitotic cell divisions, TGCs undergoing endoreplication are insensitive to genotoxic stress induced apoptosis [31], [52]. This distinction is not due to differences in genome duplication, because the nuclear genome in TGCs is uniformly and integrally duplicated, and the transcriptome contains a robust expression of S-phase genes [53]. Rather it is due to the acquisition of molecular mechanisms that prevent DNA damage or stalled replication forks from inducing apoptosis during endocycles [54], mechanisms that also allow cancer cells to resist DNA damage induced apoptosis [55].

Analyses of TSCs [31], [52], embryonic stem cells [31], and cancer cells [56] suggested that down-regulation of p53 protein expression in TGCs together with up-regulation of p21 protein expression resulted in suppression of CHK1 expression and reduced sensitivity to genotoxic stress. The results reported here, together with those describing maturation of bone marrow monoblasts into monocytes and subsequent differentiation into macrophages [15], [16], suggest that cytoplasmic localization of p21 is not only a hallmark of cancer cells, but also a developmentally regulated event that is used to increase the survival of some, but not all [57] terminally differentiated cells.

In *Drosophila*, cells that are developmentally programmed to endoreplicate their genomes do not undergo apoptosis when DNA re-replication is induced experimentally, whereas cells undergoing mitotic cell division do undergo apoptosis [54]. Moreover, when these cells switch to endoreplication, they repress the apoptotic response to DNA damage independently of cell differentiation [58]. Additionally, when these cells were allowed to recover their ability to endoreplicate, the resulting polyploid cells underwent an error-prone mitosis that induced apoptosis. Thus, endoreplication appears to be linked directly to the suppression of apoptosis. In the case of TGCs, this link appears to be Akt-dependent cytoplasmic localization of p21.

There are two Akt isoforms. Akt1 phosphorylates the human and mouse p21 protein at the same locus (T145, S146 in human cells; T140, S141 in mouse cells), thereby stabilizing the protein and localizing it to the cytoplasm in a variety of human cancer cells [5]–[8] and in TGCs (this report). In addition, Akt1 phosphorylation of p21 prevents its interaction with PCNA [59]. In contrast, Akt2 does not phosphorylate p21, but it does compete with Akt1 for binding to p21 at the site where Akt1 phosphorylates p21, thereby preventing Akt1 phosphorylation of p21 and inducing its nuclear localization [4], [60]. Thus, these two Akt isoforms regulate the subcellular localization of p21 along with its associated consequences.

### Cip/Kip Proteins

TGCs express low levels of M-phase genes and high levels of genes required for placenta function, including p57 [53]. All three *CIP/KIP* genes are transcribed in trophoblast cells, but in TSCs the Chk1 kinase selectively phosphorylates p57 and p21 proteins, thereby targeting them for ubiquitin-dependent degradation [30]. In response to FGF4-deprivation of TSCs, Chk1 expression is suppressed, which results in the expression of p57 and p21 proteins concomitant with differentiation into TGCs. Although p27 is expressed in TGCs as well as in TSCs (Figs. 1–2; [30], [31]), the requirement for p57 during placentation cannot be carried out by p27 [61]. This is consistent with the fact that p57 is essential for initiation and maintenance of endoreplication in TGCs and for maintaining TGCs in a nonproliferative state [30], [31], [33]. Nevertheless, p27 is expressed in the nucleus of TSCs (Fig. 1), and both p27 and p57 proteins are expressed in the nucleus of G-phase TGCs (Fig. 2). Therefore, the role of p27 in both the mitotic cell cycles and endocycles of trophoblast cells appears to be the same as its role in all eukaryotic cells; p27 or a p27 ortholog prevents premature activation of the CDK activity that is essential for initiation of S phase [41].

The fact that p27 is expressed at comparable levels in both TSCs and TGCs (Fig. 5B; [31]) suggests that p27 plays the same role during multiple rounds of endoreplication (endocycles) as it does during mitotic cell cycles. Although this function is not essential for mammalian development, because mice lacking a functional p27 gene do not exhibit any gross developmental defects, adults lacking p27 do have an enlarged body, manifest abnormal enlargement of their organs, and frequently develop pituitary tumors [40], [62], [63]. Therefore, although p27 is required to produce a normal healthy adult, p27 and p57 are structurally and functionally quite similar and to some extent functionally redundant.

The p27 protein is expressed ubiquitously throughout mouse development, whereas expression of the p57 protein is confined primarily to maintaining either terminal cell differentiation [64] or hematopoietic stem cell quiescence [65], [66]. Embryos lacking p57 exhibit multiple developmental abnormalities, including placentomegaly and dysplasia of placenta trophoblast cells [67]. Replacing the endogenous p57 gene with the p27 gene driven by the p57 promoter revealed that p27 could substitute for many, but not all, of the roles performed by p57 [42], [65], [66]. One exception is placenta development; p57 is essential for normal placenta development where it is among the most abundantly expressed protein-coding genes in TGCs [53]. Differences between the phenotypes of p27−/− and p57−/− mice appear to result primarily from differences in the spatial and temporal expression patterns of the two proteins, as well as differences in the sensitivity of various tissues to the level of CDK inhibition. In fact, immuno-precipitation of p27 from extracts of either TSCs or TGCs did not co-precipitate Cdk1, whereas immuno-precipitation of either p57 or p21 from TGCs did [31]. Moreover, triggering TSC differentiation by selective inhibition of Cdk1 with the chemical inhibitor RO3306 did not up-regulate p57, but endocycles occurred nevertheless [31]. Apparently, p27 is sufficient to regulate endocycles as it does in mitotic cell cycles, but p57 is critical to prevent mitosis.

As for the p21 protein, the results presented here reveal that p21 plays the same role in TGCs as it does in human cancer cells. In human cancer cells, Akt1 phosphorylates p21 at T145 and S146, thereby preventing its binding to PCNA (the processivity cofactor for DNA polymerases δ and ε), decreasing its inhibitory effect on cyclin CDK complexes, increasing p21 protein stability, localizing it to the cytoplasm, and protecting cells from apoptosis [5], [8], [39]. In mouse TGCs, Akt1 phosphorylates p21 at T140 and S141 (Fig. 5A, B, D). These sites are homologous to T145 and S146 in human p21, and they are adjacent to three amino acids that are essential for p21 nuclear localization (Fig. 5A). Cytoplasmic localization of p21 could also result from the unique ability of the p21 nuclear localization signal to bind a cytoplasmic retention protein, such as Brap2 [16], [49]. The role of cytoplasmic p21 in TGCs is to suppress apoptosis in response to DNA damage (Figs. 8–9). The anti-apoptotic properties of cytoplasmic p21 result both from binding to and inhibiting the activities of pro-apoptotic proteins [15], [68]–[70] and from inhibiting up-regulation of pro-apoptotic genes [12], [71]. In addition to its anti-apoptotic role, p21 may also constitute a feedback loop that facilitates suppression of Chk1 protein [56].

### Three Protein Kinases that Govern TSC Differentiation

The results presented here also reveal that TSC differentiation is governed by three protein kinases: Cdk1, Chk1 and Akt1. Cdk1 activity is essential for mitotic cell cycles; selective inhibition of Cdk1 in TSCs triggers endoreplication and differentiation into TGCs [31]. During mitotic cell cycles, Chk1 kinase is activated in response to DNA damage and stalled replication forks. Activated Chk1 prevents entrance into mitosis by inhibiting Cdc25, the protein phosphatase that converts the inactive form of Cdk1 into the active form that binds cyclins A or B and triggers mitosis. Independently of its role in the DNA damage response, Chk1 prevents expression of p57 and p21 proteins in TSCs in response to FGF4 mitogen stimulation (the ‘G2 restriction point’ [30]), thereby preventing TSCs from exiting the mitotic cell cycle and differentiating into TGCs. In the absence of FGF4, Chk1 expression is suppressed, thereby up-regulating p57 and p21 protein expression as TSCs differentiate into TGCs. The p57 protein inhibits Cdk1 activity, thereby preventing entrance into mitosis, which triggers endoreplication. TSCs have both p57-dependent and p57-independent pathways to trigger differentiation into TGCs [31]. In addition, FGF4 deprivation of TSCs induces up-regulation of Akt1, which can phosphorylate all three Cip/Kip proteins, and which is required for cytoplasmic localization of p21.

Remarkably, both Chk1 and Akt1 phosphorylate the same amino acids in p21, but the fate of the phosphorylated protein is cell-type specific. Phosphorylated p21 in TSCs is targeted for ubiquitin dependent degradation [30], whereas phosphorylated p21 in TGCs is stable and localized to the cytoplasm (Figs. 3–6). The reasons for this cell-type specificity remain to be elucidated. Nevertheless, they have important consequences. Whereas the effects of cytoplasmic p21 are detrimental to adults, because they promote tumor growth, they are benign in terminally differentiated cells such as TGCs and monocytes, because they promote viability in the absence of proliferation.

## Materials and Methods

### Cell Lines and Reagents

Wild-type, *p21−/−* and *p57−/−* TSC lines were previously derived and characterized [31] and were cultured as previously described [30], [31]. TSC differentiation was induced by deprivation of conditioned medium supplemented with FGF4 and heparin (referred as “FGF4 deprivation”). HEK-293T cells, NIH3T3 fibroblasts (ATCC), and primary mouse embryonic fibroblasts (PMEF, EMD Millipore) were cultured in Dulbecco’s modified Eagle’s medium (DMEM; Invitrogen) supplemented with 10% fetal bovine serum. Reagents included FGF4 (Sigma-Aldrich), etoposide (Sigma-Aldrich, E1318), Akt inhibitor-X (Santa Cruz CAS 925681-41-0), and bromodeoxyribouridine (BrdU labeling kit, Roche #11296736001). Antibodies included p21 (Santa Cruz sc-397; sc-6246), Thr145-P-p21 (sc-20220), p27 (sc-776, cell signaling #3686), p57 (GeneTex GTX62720), cytokeratin endo-A (Developmental Studies Hybridoma Bank, TROMA-1), γH2AX (Ser139) (Cell Signaling #9718), Chk1 (sc-8408), Akt1 (sc-1618), Myc-tag (Cell Signaling #2276), Cdk1 (sc-54,), Cdk2 (sc-6248), actin (sc-1616), and tubulin (DSHB, Iowa E7). 5-Ethynyl-2′-deoxyuridine (EdU) and Click-IT reaction cocktail with Alexa Fluor 594 (Invitrogen) were used according to the manufacturer’s instructions. Protein sequences were *Mus musculus* p57 (348 amino acid isoform 1, accession number NP001155096.1), p21 (159 amino acid cyclin-dependent kinase inhibitor 1, accession number NP001104569.1), and p27 (197 amino acid Cdkn1b, NP034005.2).

### Expression Vectors

Mouse *Cdkn1A/p21/Cip1* cDNA was amplified by PCR (Table 2) and cloned into pcDNA4/TO/*myc*-HisA (T-Rex, Invitrogen) to produce recombinant p21 proteins tagged with cMyc and His_6_ at their C-termini. NIH3T3 cells were co-transfected with pcDNA/6TR (containing the tetracycline repressor gene) and pcDNA/TO/p21-*myc*-His plasmids (ratio 4∶1), as indicated in the figure legends. Twenty-four hours after transfection, tetracycline (1 µg/ml) was added to the medium for 18 hours to induce p21 expression. Alternatively pcDNA/TO-p21-*myc*-His plasmids were transfected alone into NIH3T3. Twenty-four hours post-transfection, the cells were fixed and stained for p21 protein. For bacterial expression, wild-type and mutant p21 genes were cloned into pET-15b (Novagen) to produce N-terminal His_6_-p21 protein in *Escherichia coli*. Recombinant proteins were purified as described previously [30]. All constructs were confirmed by sequencing.

**Table 2:** Primers used for site-directed mutagenesis of the murine p21 cDNA.

p21(fwd)	CACGGATCCATGTCCAATCCTGGTGATGTCCGACCTGTTCCGCACAGGAGC
p21-WT(rev)	CACCTCGAGGGGTTTTCTCTTGCAGAAGACCAATCTGCGCTTGGAGTGATAGAAATCTGTCAGGCTGGTC
p21-T140V(rev)	CACCTCGAGGGGTTTTCTCTTGCAGAAGACCAATCTGCGCTTGGAGTGATAGAAATCTGTCAGGCTGACCTGCCT
p21-T140E(rev)	CACCTCGAGGGGTTTTCTCTTGCAGAAGACCAATCTGCGCTTGGAGTGATAGAAATCTGTCAGGCTCTCCTGCCT
p21-S141A(rev)	CACCTCGAGGGGTTTTCTCTTGCAGAAGACCAATCTGCGCTTGGAGTGATAGAAATCTGTCAGGGCGGTCTGCCT
p21-S141D(rev)	CACCTCGAGGGGTTTTCTCTTGCAGAAGACCAATCTGCGCTTGGAGTGATAGAAATCTGTCAGGTCGGTCTGCCT
p21-T140V, S141A(rev)	CACCTCGAGGGGTTTTCTCTTGCAGAAGACCAATCTGCGCTTGGAGTGATAGAAATCTGTCAGGGCGACCTGCCT
p21-T140E, S141D(rev)	CACCTCGAGGGGTTTTCTCTTGCAGAAGACCAATCTGCGCTTGGAGTGATAGAAATCTGTCAGGTCCTCCTGCCT

underlined bases are mutated.

### Analytical Methods

Western immuno-blotting and immuno-fluorescence were performed as previously described [30]. Secondary antibodies for immunofluorescence included Alexa Fluor 488 anti-mouse (Invitrogen A21042), Alexa Fluor 594 anti-mouse (A24921), Alexa Fluor 488 anti-rabbit (A11034) and Alexa Fluor 594 anti-rabbit (A24923). Images were acquired with a Nikon epifluorescence microscope (40x or 60x objectives) equipped with a Nikon Digital Camera DXM1200F and using Nikon ACT-1 software. Alternatively images were acquired with an inverted Leica TCS SP5II confocal microscope with GaAsP hybrid detectors (63x objective) using Leica Application Suite Advanced Fluorescence (LAS AF) software. After acquisition, images were processed with Image-J.

### Protein Kinase Assays

Recombinant mouse p21 proteins were purified from bacteria and tested as Akt1 (Sigma SRP-0353-10UG) substrates using an *in vitro* kinase assay previously described [30].

### shRNA Suppression of Akt1 and p27

Akt1 and p27 shRNA oligonucleotides were designed against three different sequences within their respective target RNA (Table 3) and cloned into pLKO.1-TRC (Addgene plasmid 10878, [72]). Lentivirus particles were produced into HEK-293T cells and used for transduction of TSCs and TGCs as previously described [30]. Scramble shRNA was used as a control (Addgene, plasmid 1864).

**Table 3:** Target Sequences for shRNAs.

Akt1-1	AACGGCCTCAGGATGTGGATC
Akt1-2	AAGCTGGAGAACCTCATGCTG
Akt1-3	AAGCCCCAGGTCACCTCTGAG
p27-1	ATCTCTTCGGCCCGGTCAA
p27-2	GCACTGCCGGGATATGGAA
p27-3	CGCUGGCACUGUGGAGCAG

All three shRNAs were packaged into a single lentivirus preparation.
